# MCP-1, KC-like and IL-8 as critical mediators of pathogenesis caused by *Babesia canis*

**DOI:** 10.1371/journal.pone.0190474

**Published:** 2018-01-05

**Authors:** Asier Galán, Iva Mayer, Renata Barić Rafaj, Krešo Bendelja, Velimir Sušić, José Joaquín Cerón, Vladimir Mrljak

**Affiliations:** 1 ERA Chair project ''VetMedZg'', Clinic for Internal Diseases, Faculty of Veterinary Medicine, University of Zagreb, Zagreb, Croatia; 2 Clinic for Internal Diseases, Faculty of Veterinary Medicine, University of Zagreb, Zagreb, Croatia; 3 Department of Chemistry and Biochemistry, Faculty of Veterinary Medicine, University of Zagreb, Zagreb, Croatia; 4 Institute of Immunology, Zagreb, Croatia; 5 Department of Animal Husbandry, Faculty of Veterinary Medicine, University of Zagreb, Zagreb, Croatia; 6 Department of Animal Medicine and Surgery, University of Murcia, Spain; Johns Hopkins University, UNITED STATES

## Abstract

Canine babesiosis caused by the intraerythrocytic protozoan parasite *Babesia canis* is a tick-borne disease characterized by a host response that involves both cellular and humoral immunity. This study focuses on the secretion of cytokines Granulocyte-Macrophage Colony-Stimulating Factor (GM-CSF), Keratinocyte Chemotactic-like (KC-like), Interleukins (IL)-2, IL-7, IL-8, IL-10, IL-15, IL-18 and Monocyte Chemotactic Protein-1 (MCP-1) in babesiosis caused by *Babesia canis* upon treatment with Imizol**®**. We assessed time dependent changes in cytokine levels and tested whether these changes correlate with pathogenesis of the disease. Sixteen healthy dogs and 31 dogs infected with *Babesia canis*, of which 18 showed complications, were treated with Imizol**®**. One dog died during the study (3.2%). Longitudinal study was perfomed by monitoring dogs at the first day of presentation (day 1) and 6 days later (day 7). Our results show that higher MCP-1 levels on day 1 are positively associated with the occurrence of complications, (complicated vs. uncomplicated; p = 0.00016). A similar pattern was observed for KC-like on day 1 (p = 0.0326) and day 7 (p = 0.044). Moreover, babesiosis caused by *B*. *canis* produced a steady increase in IL-8 levels with a moderate to strong negative correlation with erythrocyte counts and hematocrit in uncomplicated diseased dogs only (Spearman's rank correlation coefficient r_s_ = -0.582 and r_s_ = -0.598 respectively). Like for MCP-1, KC-like levels also differed in complicated and uncomplicated diseased dogs on day 1 (p = 0.03236) and day 7 (p = 0.044). Furthermore, KC-like levels were strongly correlated with IL-8 levels (r_s_ = 0.663–0.7) and non-segmented neutrophil counts (rs = 0.572–0.732) in both diseased groups. Analysis of ROC suggests the use of serum levels of MCP-1 and IL-7 as predictors of the occurrence of complications with an AUC of 0.906 and 0.896 respectively and linear combinations of MCP-1, KC-Like, IL-7 and GM-CSF with values up to AUC = 0.983. Cytokine cluster analysis presented in this study can contribute to a better understanding of the pathogenesis of babesiosis and serve as a prognostic tool for the early detection of cases with highest likelihood of developing complications. Overall, our studies show that infection by *B*. *canis* elicits a cytokine pattern that is distinct from that observed with *B*. *rossi*, and that some of the inflammatory mediators can be useful to predict complications. Our results also suggest targets for the development of novel therapeutic strategies in babesiosis caused by *B*. *canis*.

## Introduction

Acute babesiosis is a malaria-like infection, characterized by fever, hemolytic anemia and hemoglobinuria. In dogs, it is usually caused by *Babesia canis*, present in North America, Southern Europe, parts of Asia and Africa [1] whose vectors are ticks such as *Dermacentor reticulatus* while other species are transmitted by *Haemaphysalis leachi* (*B*. *rossi*) and *Rhipicephalus sanguineus* (*B*. *vogeli*) [2]. Babesiosis caused by *B*. *canis* usually is characterized by low parasitemia, which does not necessarily correlate with the severity of illness [3]. Hemoglobinuria has been described in naturally infected dogs [4, 5] whereas experimental infection with *B*. *canis* resulted in transient low parasitemia (1–2%), thrombocytopenia, an increase in the activated partial thromboplastin time (APTT) and hypotension [6].

In contrast, *B*. *rossi*, the main causative organism of canine babesiosis in sub-Saharan Africa, generally elicits a virulent illness with high parasitemia [7], hypoglycemia [8] and cerebral, lung and renal involvement [9] associated with mortality. Interestingly, a polymorphic phosphoprotein, *B*. *rossi* erythrocyte membrane antigen 1 (BrEMA1), expressed on the cytoplasmic membrane of *B*. *rossi*-infected erythrocytes, has been recently described. This protein is suspected to be a major virulence factor in *B*. *rossi-*induced canine babesiosis [10]. *B*. *canis* and *B*. *vogeli* do not express the BrEMA1 gene.

Other babesial species cause a broad spectrum of clinical signs: *B*. *vogeli* usually causes a subclinical to moderate clinical disease with possible severe to fatal hemolytic anemia in young dogs and pups [11]. Immune-mediated hemolytic anemia (IMHA) [12] is common in *B*. *vogeli* infection but inflammatory patterns are not so uniform as in *B*. *canis* infections [13]. A currently unnamed large form of *Babesia* was described for the first time in a dog under chemotherapy for lymphoma [14]. *B*. *gibsoni* causes frequently achronic disease often associated with weight loss and fatigue [15]. *B*. *conradae* is usually more virulent than *B*. *gibsoni* resulting in higher parasitemia, more severe anemia and higher rate of mortality [16]. *B*. *microti-like* piroplasm (*Theileria annae*) is a recently described small piroplasm causing azotemia [17].

Canine babesiosis is defined as a protozoal sepsis [18] occurring along with a generalized uncontrolled inflammatory response of the host [19] which represents a central factor in the development of complications. Infection initiates a mechanism of antibody-mediated cytotoxic destruction of erythrocytes. Autoantibodies are directed against components of the membranes of infected and uninfected erythrocytes causing intra- and extravascular hemolysis, which can evolve to anaemia. C-reactive protein (CRP) and serum amyloid A (SAA) significantly increase in canine babesiosis but their levels are not related with the occurrence of complications neither with outcome [18, 20]. Proinflammatory cytokines and chemokines, such as TNF-α, IFN-γ, IL-1β, IL-2, IL-6, IL-8, IL-12, IL-18 and MCP-1 are necessary to initiate an effective inflammatory response [21] and promote the transendothelial migration of leukocytes. TNF-α and IL-1β are considered the proximal or initiator cytokines of the proinflammatory response as they trigger the production of other cytokines such as IL-6 and IL-8 (distal cytokines). On the other hand, IL-4, IL-10 and transforming growth factor-beta (TGF-β), are required to down-regulate the cell-mediated inflammatory response by inhibiting the synthesis of pro-inflammatory cytokines [22]. In fact, the acute phase response (APR) which results in systemic inflammatory response syndrome (SIRS), one of the complications of babesiosis, is a common feature of other types of sepsis [23]. Tissue hypoxia, trigger of many of the clinical signs and studied comprehensively in *B*. *rossi* infection, is considered to be more important than hemoglobinuria, and its consequent nephrotoxic effect, as a cause of kidney damage in dogs with babesiosis [24]. In a similar way as in malaria, the inefficient use of oxygen by mitochondria triggered by the effects of inflammatory cytokines is the main cause of hypoxia-related tissue damage [25]. Release of reactive oxygen species and harmful cytokine effects have been linked to endothelial damage and augmented vascular permeability in canine babesiosis [26, 27, 28].

Uncomplicated babesiosis might be a consequence of hemolysis without excessive inflammatory response while in complicated canine babesiosis and in malaria caused by *Plasmodium falciparum* pathology is believed to be the result of excessive production of pro-inflammatory cytokines [29] generating SIRS and multiple organ dysfunction syndrome (MODS) [30] with a 15% mortality rate.

In case of the most severe complications of *P*. *falciparum* malaria, cerebral malaria (CM) and severe malarial anemia (SA), both likely occur along with a dysregulation of the immune system [31]. Cytokines in malaria are key factors in regulating the progression of disease and are closely related to symptoms, parasitemia, severity of pathology and outcome [32]. High concentrations of pro-inflammatory cytokines such as TNF-α, IFN-γ, IL-6, IL-8, IL-18 and MCP-1 have been associated with severe malaria and death [33]. Regulatory cytokines such as IL-10 and TGF-β are important to limit that pro-inflammatory response [34].

Despite the comprehensive research on the inflammatory response caused by babesiosis, it is not completely understood how imbalances in cytokine levels develop and determine the course of the disease, occurrence of complications and outcome. In this study we use a multiplex approach to monitor simultaneously the concentrations of diverse cytokines and detect expected imbalances, to determine which cytokines discriminate more significantly uncomplicated and complicated babesiosis and to establish levels of cytokines as prognostic markers.

## Materials and methods

### Animals

47 dogs were retrospectively included in this study and divided into 2 main groups. **Group 1**consisted of 31 dogs naturally infected by *B*. *canis*, admitted at the Clinic for Internal Diseases, Faculty of Veterinary Medicine, University of Zagreb, Croatia, with clinical signs of acute babesiosis. The clinical manifestations included anorexia, lethargy and fever, pale mucous membranes, anemia, jaundice, hemoglobinuria or hematuria, splenomegaly, tachycardia and vomiting. Dogs were of various breeds, between 1 and 14 years of age, 18 males and 13 females. Protocol was approved by the Ethics Committee for Animal Experimentation, Faculty of Veterinary Medicine, University of Zagreb, Croatia (Permit No: 251–61–01/139–12–2). Permission to collect blood samples was obtained from each dog owner. Owners were informed about the use of the specimens and the aims of the research.

Diagnosis was confirmed by demonstration of the parasites within the infected erythrocytes in Romanowsky-stained thin blood smears. One dose (6 mg/kg of body weight) of imidocarb dipropionate (Imizol®, Schering–Plough) was administered to all the dogs subcutaneously on the day of admission (day 1). Additional treatment consisted of various fluids (colloid and crystalloid therapy), and whole blood transfusion when it was indicated. Subspecies were confirmed using PCR (polymerase chain reaction) [6]. On the basis of clinical manifestations and laboratory data the infected dogs were classified into two subgroups: complicated (18 dogs, 8 Female, 10 Male) and uncomplicated (13 dogs, 5 Female, 8 Male).The classification of clinical manifestations of the complicated form of babesiosis is based on the World Health Organization (WHO) classification for malaria [35]. The main complications are the development of an excessive inflammatory response named”systemic inflammatory response syndrome” or SIRS [36] and a multiple organ dysfunction syndrome or MODS [37].

According to the criteria for the diagnosis of SIRS used in this study [9], [38] dogs were classified as SIRS positive if two or more of the following 4 criteria were fulfilled: body temperature higher than 39.5°C or lower than 38°C, heart rate more than 160 beats/min, respiration rate more than 20 breaths/min and WBC count less than 6 × 10^9^/L or more than 12 × 10^9^/L or with more than 10 percent band neutrophils. Dogs with obvious concurrent inflammatory processes as trauma, wounds or infections, known cardiopathies or neoplastic diseases were excluded from the study. Dogs treated with any anti-inflammatory medication within 3 weeks prior to diagnosis were also excluded. All dogs showing complications were treated according to standard procedures (infusion, anti-inflammatories and blood transfusion only in case hematocrit was under 20%). Blood samples for cytokine quantification were obtained 6–8 hours after the treatment was administered.

An animal was classified as MODS positive if two or more of the following criteria were fulfilled: renal dysfunction (serum creatinine concentration higher than 180 μmol/l), hepatic dysfunction (both alanine aminotransferase (ALT) greater than 176 IU/l and alkaline phosphatase (AP) greater than 360 IU/l), respiratory system dysfunction (radiographic evidence of pulmonary oedema or dyspnoea), and muscular involvement (creatine phosphokinase (CPK) more than 600 IU/l) [9]. Cases with high serum creatinine concentration due to pre-renal causes (e.g., dehydration due to vomiting) were excluded after examining oral mucosa, skin turgor and ocular appearance as well as checking concentrations of serum urea, serum albumin and total protein and determining urine specific gravity. 66% of complicated cases were MODS positive and all of them were SIRS positive.

**Group 2** (healthy controls) included two subgroups: animals treated by prophylactic single application of imidocarb dipropionate (Imizol®, Schering-Plough) consisted of 8 dogs (**Group 2a**) of different breeds and sexes, aged from 1 to 10 years. These dogs were considered healthy based on physical examination and hematological and biochemical data, and they attended the hospital to receive a prophylactic dose of imidocarb dipropionate (6 mg/kg) as a preventive measure against babesiosis upon owners’ request. Another subgroup (**Group 2b**) consisted of 8 dogs, different breeds and genders, aged from 1 to 14 years. These dogs attended routine check controls and were considered healthy based on clinical examination and hematological and biochemical data. Due to the fact that no significant differences in clinical hematology, serum biochemistry and cytokine profile results were identified between the two subgroups, these subgroups were merged into a single healthy control group.

All serum samples from dogs were screened for simultaneous qualitative detection of circulating antibodies, both immunoglobulin G (IgG) and IgM, to *B*. *canis*, *Borrelia burgdorferi*, *Anaplasma phagocytophilum* and *Dirofilaria immitis* antigen using an in-clinic enzyme-linked immunosorbent assay (ELISA) SNAP 4Dx (IDEXX Laboratories, Hoofddorp, The Netherlands), according to the manufacturer’s instructions.

### Blood samples

Blood samples from **groups 1 and 2a** were collected from the cephalic vein on the day of admission, and 6 days after the administration of imidocarb dipropionate (Imizol®). Blood samples for analysis from **group 2b** were obtained during routine visits to a Clinic. Blood was collected using a 20 gauge needle in tubes with EDTA for hematological analysis and serum vacutainer tubes. A complete list of all samples with cytokine concentration values and hematologic parameters as well as reference ranges is included in S1 Table.

### Hematological analysis

Blood smears were prepared and PCR reactions performed using blood obtained on EDTA. White blood cell count (WBC), platelet count, hematocrit (HCT) and other hematologic parameters were determined using a Horiba ABX automatic hematology analyser (Diagnostics, Montpellier, France). Segmented and non-segmented neutrophils were manually counted in blood smear by experienced personnel.

### Quantification of cytokines in serum

Blood collected in serum tubes was allowed to clot and tubes were centrifuged at 1200 xg, 10 minutes at room temperature. A portion of the serum was used for routine biochemical analysis while the remainder was stored at −80°C until it was used to analyse cytokines. Cytokine profiles were determined using the MILLIPLEX MAP Canine Cytokine/ Chemokine Magnetic Bead Panel (CCYTO-90K Millipore, Billerica, MA) with an automated analyser (Luminex 200, Luminex Corporation, Austin, TX). The concentrations of interleukin-2 (IL-2), IL-7, IL-8, IL-10, IL-15, IL-18, MCP-1, granulocyte-macrophage colony stimulating factor (GM-CSF) and keratinocyte chemoattractant KC-like were analyzed. Prior to analysis, samples were thawed, vortexed, and centrifuged at 1000x g for 10 min to separate particulates. The analytes were measured in duplicate, according to the manufacturer’s instructions. Briefly, the plate was pre-wet with wash buffer and standards, controls and samples were added to the appropriate wells. After washing, the detection antibodies were added to each well. After incubation, the plate was run on Luminex. The median fluorescent intensity (MFI) was analyzed and the concentrations of analytes were derived using standard curves.

### Statistical analysis

Statistical analysis was performed using STATISTICA version 10 (StatSoft. Inc., 2011). Given that most variables were not normally distributed, statistical differences were assessed using the non-parametric Mann-Whitney *U* test. p-values of <0.05 were considered to be significant. Correlations were assessed by both Spearman's rank correlation test and Pearson's product-moment correlation test.

## Results

We have analysed cytokine concentrations of 31 dogs suffering from babesiosis versus 16 healthy animals (Table 1). We observed higher levels of cytokines on day 1. This trend reached a plateau or reverted (remarkably for IL-10) on day 7 for most cytokines with the exception of IL-8 for which an increase was noted.

**Table 1:** 

Group	Gender	Age (years) Average±Standard deviation
**Group 1**		
**Diseased uncomplicated (n = 13)**	8M (61.5%) 5F (38.5%)	4.31±2.81
**Diseased complicated (n = 18)**	10M (56%) 8F (44%)	5.83±3.56
**Group 2 (a+b)**		
**Healthy (n = 16)**	9M (56%) 7F (44%)	5.07±3.78

All cytokine levels shown registered a significant increase upon babesiosis (Table 2) on day 1 and all except IL-7, IL-10 and MCP-1 keep significantly higher levels than healthy controls on day 7. When comparing uncomplicated and complicated diseased dogs, all cytokines except IL-8, IL-10, IL-15 and IL-18 are significantly different on the day 1 but only KC-like and MCP-1 show significantly different levels on day 7. The most significant longitudinal variation within uncomplicated cases was for IL-10, IL-8 and GM-CSF while only IL-10 and IL-8 varied significantly over time in complicated cases.

**Table 2:** Cytokine levels in different groups and subgroups. Cytokine concentrations (in pg/ml serum) for healthy dogs (n = 16), *B*. *canis*- infected dogs on the day 1 (n = 31) and 7 (n = 30) and subset of uncomplicated (n = 13) and complicated groups (n = 18). 1 dog with complications died during the study. p-values for significant differences are shown for each comparison.

	CONCENTRATION (pg / ml serum)								
	GM-CSF	KC-like	IL-2	IL-7	IL-8	IL-10	IL-15	IL-18	MCP-1
**Healthy (n = 16)**									
Min-Max	60–154	28–269	10–75	39–109	122–235	1–1	42–142	2–840	159–792
Median	87	39	17	55	137	1	77	83	193
SD	24	58	26	20	42	0	26	232	154
**Diseased day 1 (n = 31)**									
Min-Max	130–782	104–2739	22–2514	31–1957	10–6502	1–2106	12–5436	2–2278	163–1921
Median	182	532	96	101	856	560	160	188	390
SD	176	639	525	378	1541	421	1032	487	494
p-value	< 0.0001	< 0.0001	< 0.0001	0.019	< 0.0001	< 0.0001	0.007	0,044	0,001
**Diseased day 7 (n = 30)**									
Healthy(n = 16)									
Min-Max	135–2822	110–2184	12–4038	35–3633	425–8818	1–548	29–5548	2–8748	156–7667
Median	196	561	49	80	2754	1	219	207	237
SD	544	602	774	706	2185	108	1089	1900	1434
p-value	< 0.0001	< 0.0001	0.014	0.1308	< 0.0001	0.321	0.007	0.029	0.09
**Uncomplicated day 1 (n = 13)**									
Min-Max	130–220	104–969	22–187	31–119	35–5019	1–2106	12–342	2–490	163–436
Median	143	330	89	51	601	461	111	145	224
SD	29	282	44	26	1477	527	110	180	95
**Complicated day 1 (n = 18)**									
Min-Max	131–782	162–2739	22–2514	35–1957	10–6502	180–1499	12–5436	2–2278	181–1921
Median	254	567	131	139	908	592	265	188	576
SD	198	749	665	470	1620	342	1324	610	552
p-value	0.00066	0.0322	0.025	0.00019	0.509	0.675	0.065	0.34683	0.00015
**Uncomplicated day 7 (n = 13)**									
Min-Max	139–272	117–2009	12–276	39–183	1391–6294	1–1	29–1109	2–762	156–282
Median	154	440	28	68	2852	1	184	145	191
SD	44	474	87	48	1362	0	309	231	42
**Complicated day 7 (n = 17)**								
Min-Max	135–2822	110–2184	12–4038	35–3633	425–8818	1–548	29–5548	2–8748	156–7667
Median	254	830	65	84	1911	1	253	225	365
SD	689	635	1005	917	2695	141	1406	2484	1844
p-value	0.059	0.04455	0.391	0.503	0.503	0.601	0.769	0.834	0.013

**Fig 1:**
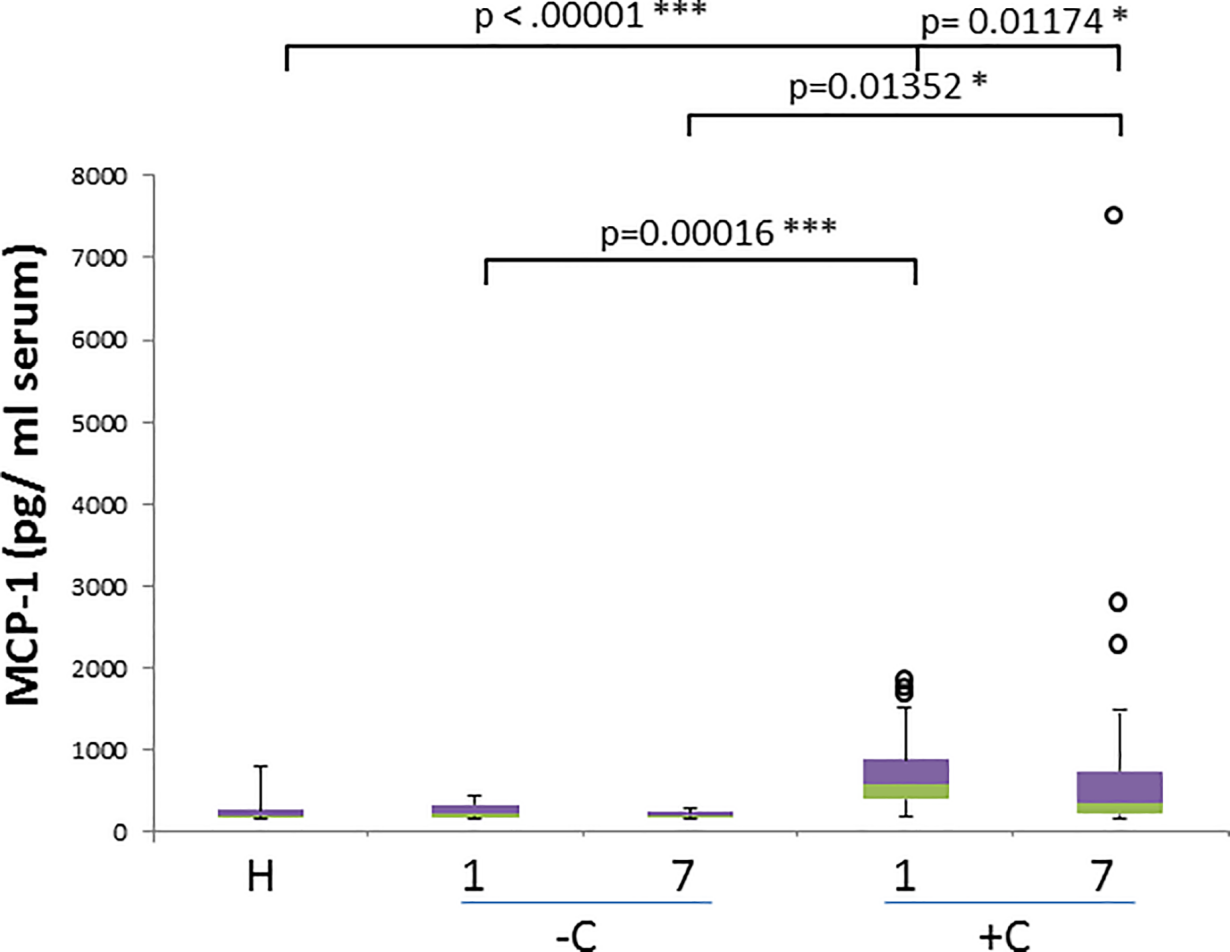
MCP-1 concentration in healthy and naturally infected dogs and its relation with outcome. Box and whisker plot with outliers showing interquartile range (median bar in intersect between two colors in box) of MCP-1 concentration values among different groups: healthy dogs (H), uncomplicated (-C) and complicated (+C) cases for both day 1 and day 7. Mann-whitney test p- values are shown for comparison of different groups.

MCP-1 concentrations were significantly elevated for the *B*. *canis*-infected dogs when comparing healthy dogs and complicated diseased dogs on the day 1 (p<0.00001) and 7 (p = 0.01174). Uncomplicated and complicated cases on both day 1 and 7 (p = 0.00016 and p = 0.01352 respectively) show significant differences in MCP-1 levels (Fig 1).

**Fig 2:**
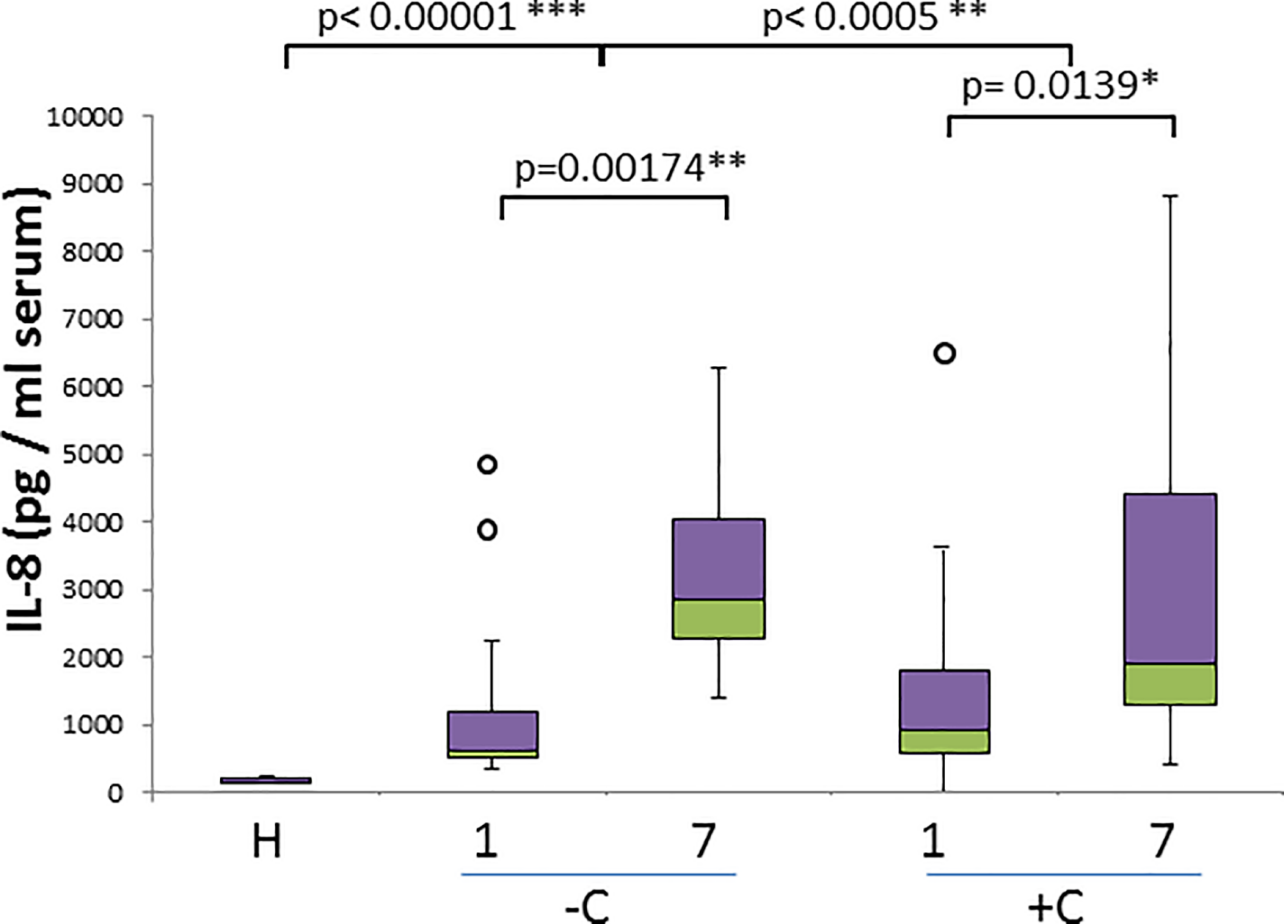
IL-8 concentration in healthy and naturally infected dogs and its relation with outcome. Box and whisker plot with outliers showing interquartile range (median bar in intersect between two colors in box) of IL-8 concentration values among different groups among different groups: healthy dogs (H), uncomplicated (-C) and complicated (+C) cases for both day 1 and day 7. Mann-whitney test p- values are shown for comparison of different groups.

We observed a very significant increase in IL-8 concentration (Fig 2) comparing healthy dogs with uncomplicated and complicated cases (p< 0.00001 and p< 0.0005 respectively) with even more significant increase on day 7 (Fig 2). IL-8 levels are not useful to discriminate uncomplicated and complicated cases, but increased over time in both groups (p = 0.00174 for uncomplicated; p = 0.0139 for complicated).

**Fig 3:**
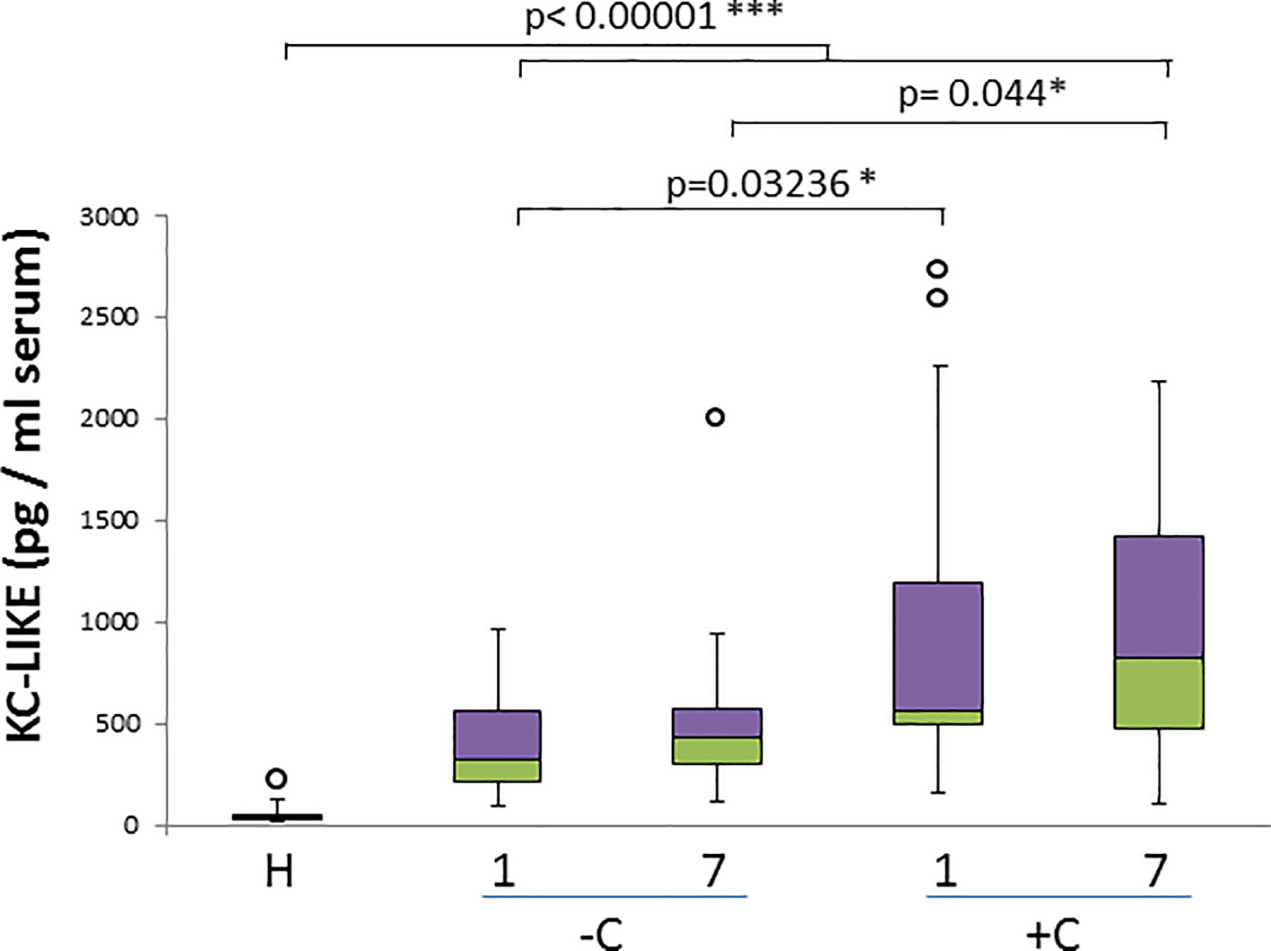
KC-like concentration in healthy and naturally infected dogs and its relation with outcome. Box and whisker plot with outliers showing interquartile range (median bar in intersect between two colors in box) of KC-like concentration values among different groups: healthy dogs (H), uncomplicated (-C) and complicated (+C) cases for both day 1 and day 7. Mann-whitney test p- values are shown for comparison of different groups.

KC-like levels show a very significant increase in all diseased animals in both day 1 and day 7 (p < 0.00001). Additionally, KC-like levels show significant differences when comparing uncomplicated and complicated cases both on day 1 and 7 (p = 0.0324 and p = 0.044 respectively) (Fig 3) (Table 2). We registered a strong negative correlation of KC-Like levels with erythrocyte counts (r_s_ = -0.627) for complicated cases on day 7, and a positive correlation with non-segmented neutrophil percent for both diseased groups on the same day (r_s_ = 0.732 for uncomplicated, r_s_ = 0.736 for complicated) as well as in complicated cases on the day 7 (r = 0.572) (Table 3).

**Table 3:** Highest Spearman' s rank and Pearson's product moment correlation coefficients (r_s_ and r). Highest values for r_s_ and r (in brackets) for cytokine:cytokine and cytokine:hematologic parameter within different groups. E: Erythrocyte count, HCT:Hematocrit, Thr: Thrombocyte count, NN: Non-segmented neutrophil count SN: Segmented neutrophil count, L: Leukocyte count, Eos: Eosinophil count.

**Cytokine: cytokine**	**GM-CSF**	**IL-2**	**IL-7**	**IL-8**	**IL-15**	**IL-18**	**MCP-1**
**Healthy**							
IL-7						0.482(0.769)	0.716(0,782)
IL-18							0.498(0.895)
**-C day 1**							
GM-CSF			0.634(0.722)				
KC-LIKE				0.7(0.866)			
**+C day 1**							
KC-LIKE				0.663(0.654)			
IL-2			0.717(0.798)				
**-C day 7**							
GM-CSF		0.969(0.827)	0.848(0.796)				0.806(0.810)
KC-LIKE				0.692(0.777)			
IL-2			0.822(0.726)		0.65(0.832)		0.837(0.793)
IL-7						0.717(0.742)	
**+C day 7**							
GM-CSF		0.779(0.934)	0.853(0.973)		0.751(0.956)	0.771(0.958)	0.746(0.946)
IL-2			0.810(0.981)		0.799(0.984)		0.500(0.891)
IL-7					0.893(0.993)		0.597(0.917)
IL-15						0.796(0.914)	0.525(0.827)
**Cytokine:Hematology**	**E**	**HCT**	**Thr**	**NN%**	**SN%**	**Eos**	**Mono%**
**Healthy**							
GM-CSF						0.563(0.758)	
MCP-1	-0.611(-0.572)		0.578(0.597)				
**-C day 1**							
KC-LIKE				0.732(0.812)			
IL-8	-0.582(-0.863)	-0.598(-0.542)		0.514(0.677)			0.641(0.825)
**+C day 1**							
KC-LIKE				0.736(0.834)			
**+C day 7**							
GM-CSF						0.563(0.796)	
KC-LIKE	-0.627(-0.656)	-0.648(-0.688)		0.572(0.610)			
IL-7						0.546(0.766)	
IL-15						0.513(0.746)	
IL-18						0.612(0.880)	

**Fig 4:**
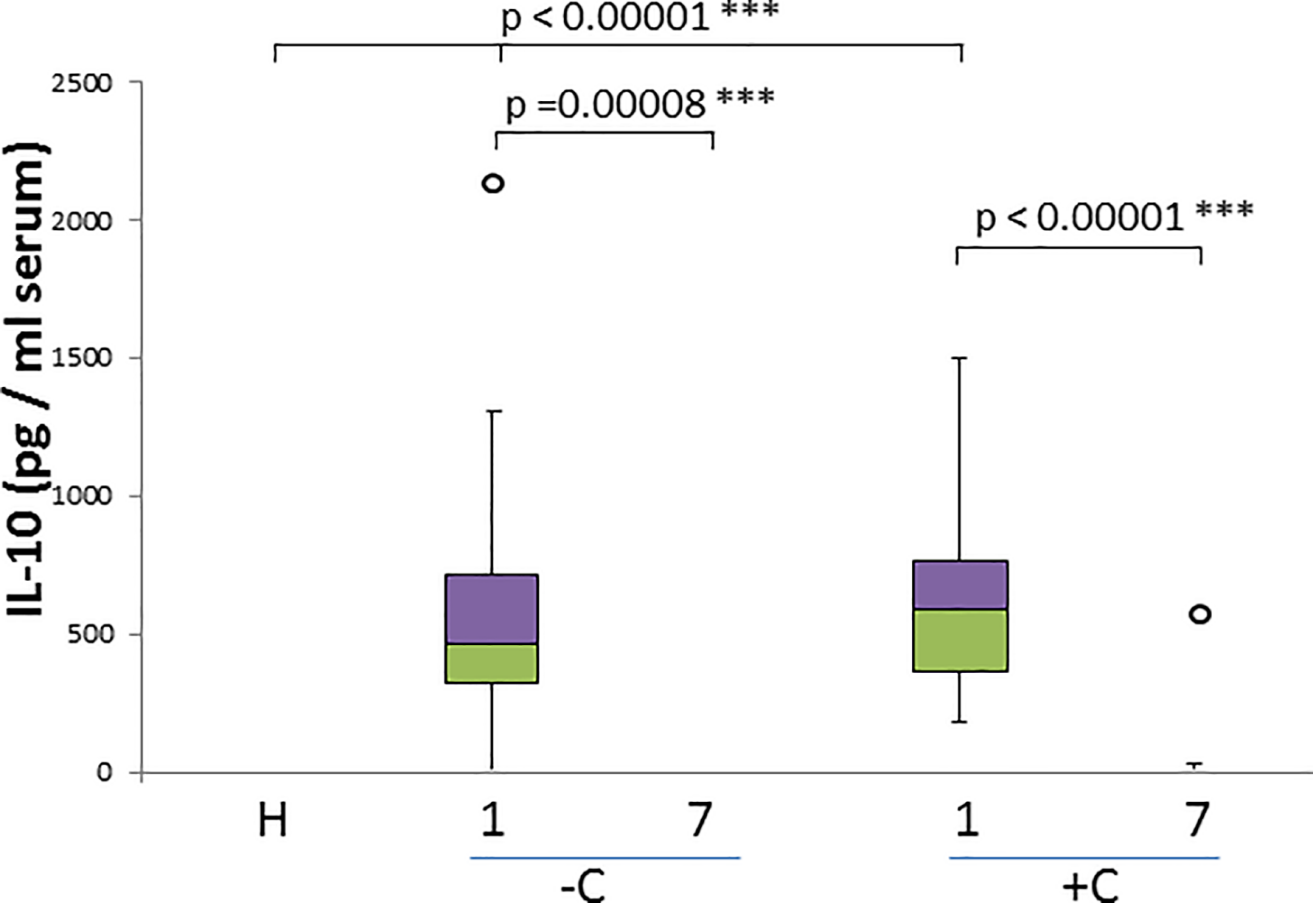
IL-10 concentration in healthy and naturally infected dogs and its relation with outcome. Box and whisker plot with outliers showing interquartile range (median bar in intersect between two colors in box) of IL-10 concentration values among different groups: healthy dogs (H), uncomplicated (-C) and complicated (+C) cases for both day 1 and day 7. Mann-whitney test p- values are shown for comparison of different groups.

We registered a very significant IL-10 increase in all diseased animals (p<0.00001) on day 1 (Fig 4) and a decrease back to the levels registered in healthy dogs on the day 7 of treatment (slightly more significant in complicated cases with a p<0.00001 vs. a p = 0.00008 for uncomplicated cases). No association with the occurrence of complications was detected (Fig 4).

Interestingly, IL-8 data obtained on the day 1 of treatment show a negative correlation with both hematocrit (r_s_ = -0.598) and erythrocyte count (r_s_ = -0.582) in uncomplicated cases on the day 1. Additionally, IL-8 shows a very strong (r = 0.7) correlation with KC-Like among uncomplicated cases in measurements performed on day 1, as well as in the same group on day 7 (r = 0.692) (Table 3) confirming results obtained previously for *B*. *canis*-caused babesiosis [39].

**Fig 5:**
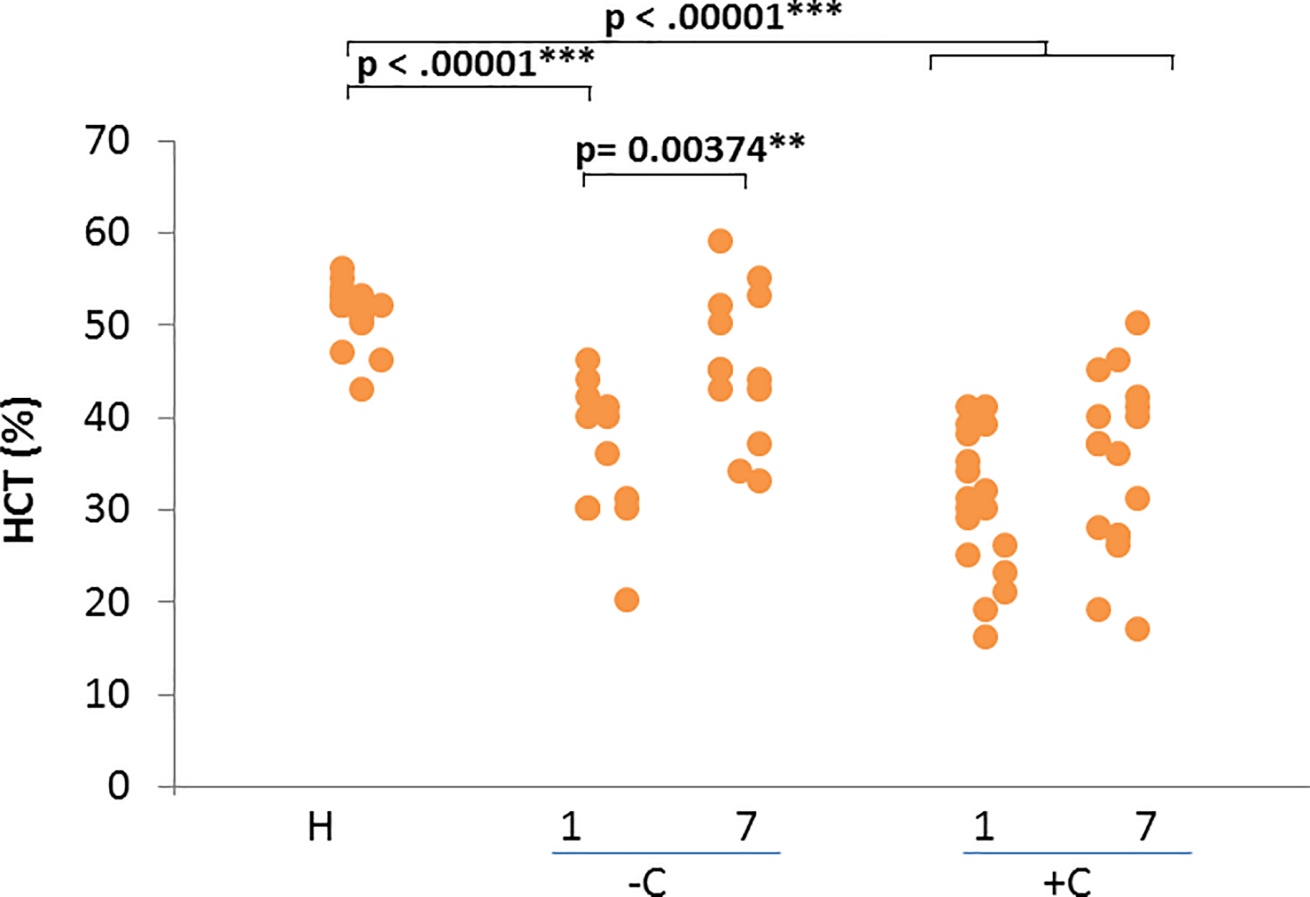
Hematocrit (HCT) values and outcome. From left to right hematocrit values for healthy dogs and values registered on day 1 and 7 of treatment for uncomplicated (-C) and complicated cases (+C). Mann-whitney test p- values are shown for comparison of different groups.

HCT is significantly decreased (p< 0.0001) in both diseased groups on day 1 but HCT normalized on day 7 in uncomplicated cases only (Fig 5).

**Fig 6:**
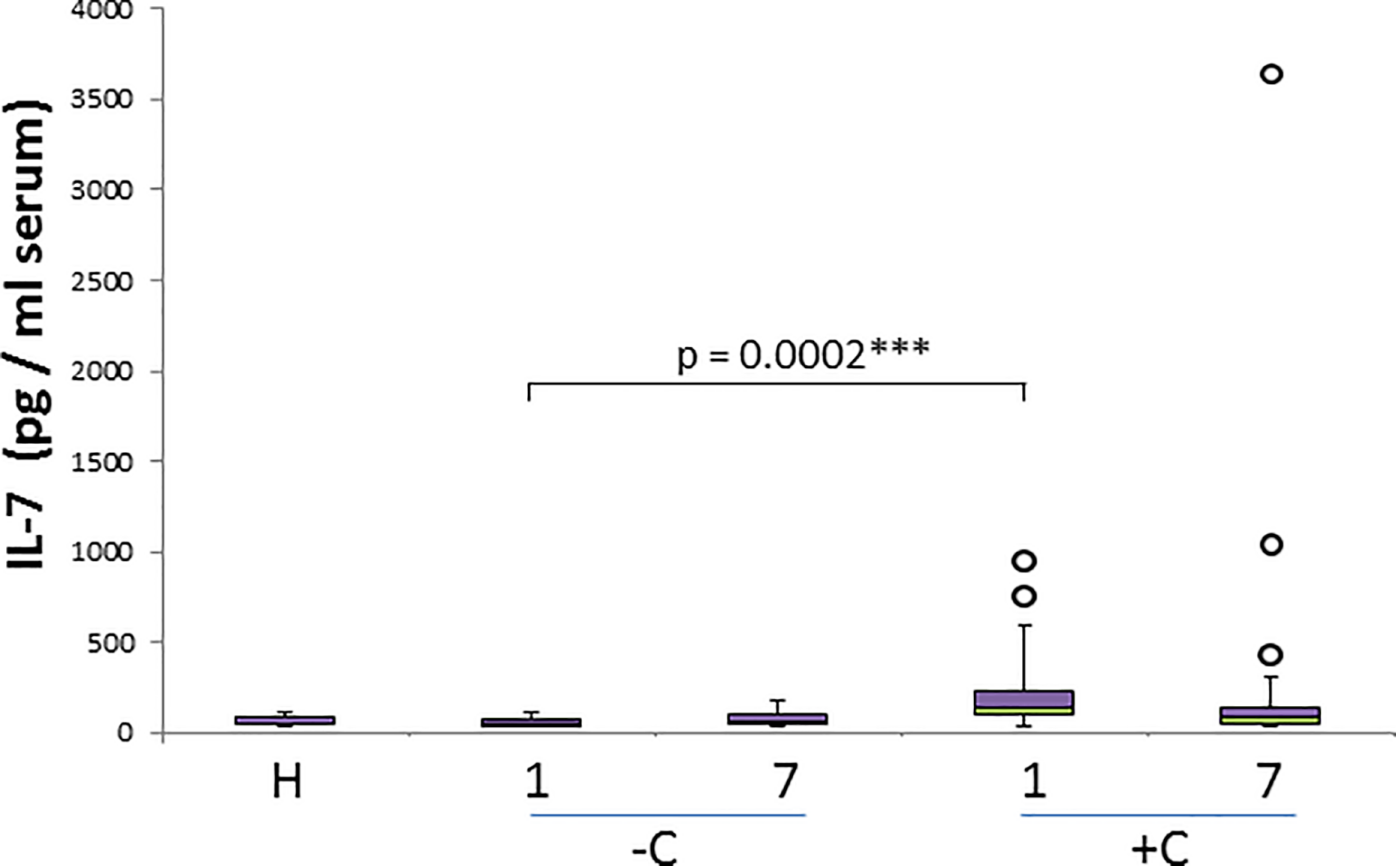
IL-7 concentration in healthy and naturally infected dogs and its relation with outcome. Box and whisker plot with outliers showing interquartile range (median bar in intersect between two colors in box) of IL-7 concentration values among different groups: healthy dogs (H), uncomplicated (-C) and complicated (+C) cases for both day 1 and day 7. Mann-whitney test p- values are shown for comparison of different groups.

IL-2 and IL-7, as the rest of cytokines analysed, show a significant increase on the day 1 (Table 2), with significantly higher concentrations in complicated cases. IL-7 shows significant differences comparing uncomplicated and complicated cases (p = 0.0002) (Fig 6), whereas IL-2 shows lower significance for the differences between both groups (p = 0.0251). None of these 2 cytokines are able to discriminate uncomplicated and complicated cases on day 7.

**Fig 7:**
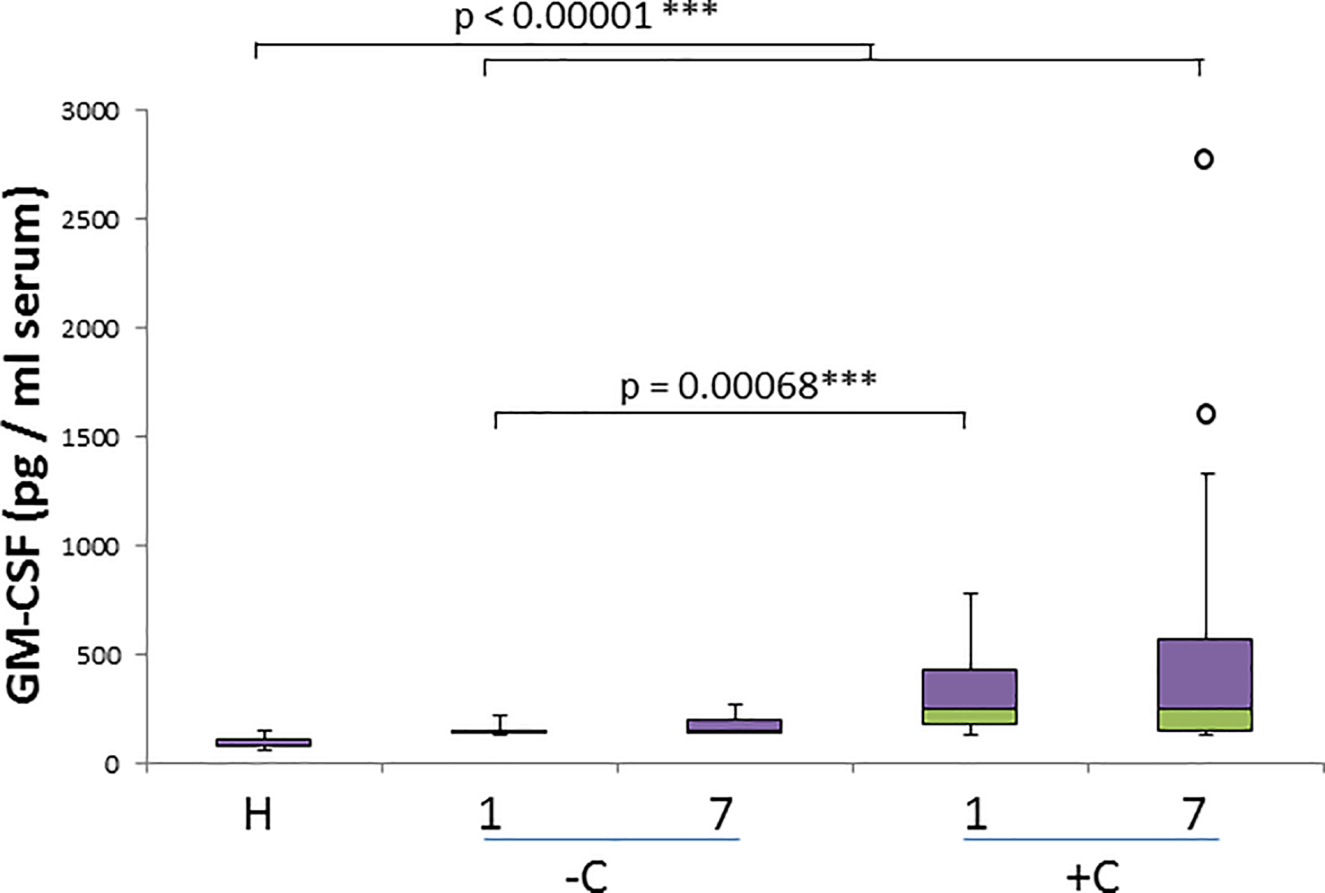
GM-CSF concentration in healthy and naturally infected dogs and its relation with outcome. Box and whisker plot with outliers showing interquartile range (median bar in intersect between two colors in box) of GM-CSF concentration values among different groups: healthy dogs (H), uncomplicated (-C) and complicated (+C) cases for both day 1 and day 7. Mann-whitney test p- values are shown for comparison of different groups.

GM-CSF discriminates very significantly (p< 0.00001) healthy dogs and all diseased groups (Fig 7), most notably when comparing healthy dogs with records obtained on day 7 of treatment (Table 2). There is a significant difference in GM-CSF concentrations for uncomplicated and complicated cases (p = 0.00068) on the day 1 but no significant differences were found on day 7. Additionally, GM-CSF shows the highest r_s_ of all cytokines with IL-2 and IL-7 on day 7, both in uncomplicated (r_s_ = 0.969 and 0.848) and in complicated cases (r_s_ = 0.779 and 0.853) (Table 3).

Other cytokines (IL-15 and IL-18) show significantly elevated levels in diseased animals but no significant association with complications (Table 2).

**Fig 8:**
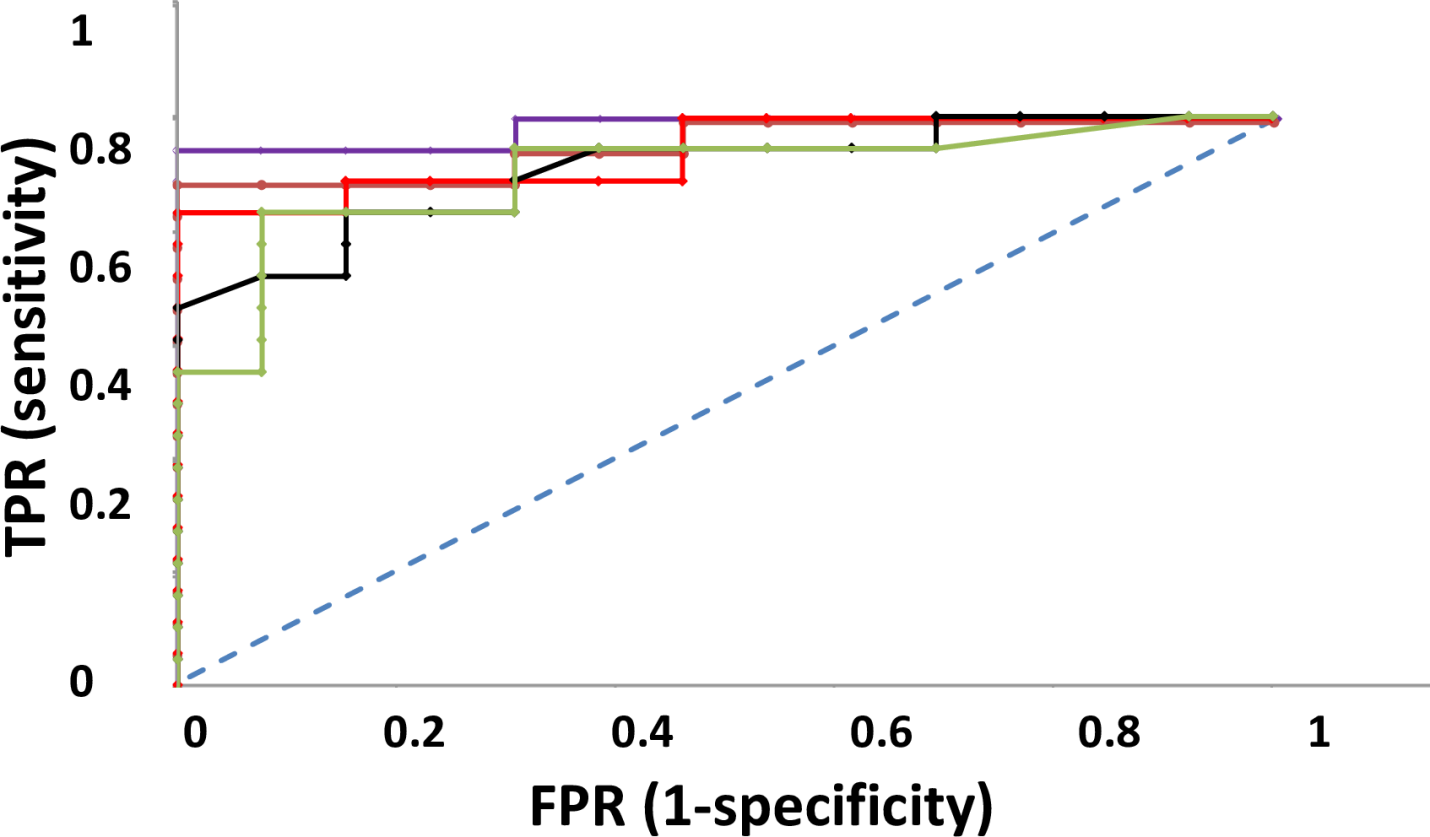
Receiver operating characteristic (ROC) curve as a tool for prognosis of complications in *B*.*canis-*caused babesiosis and cut-off values. ROC plot showing **c**urves corresponding to individual cytokine values and linear combinations of different cytokine clusters for uncomplicated vs. complicated cases. MCP-1 (black), IL-7 (green), LC1 = KC-Like+IL-7+2MCP-1(red), LC2 = KC-Like+2IL-7+2MCP-1 (orange), LC3 = GM-CSF+KC-Like+2IL-7+2MCP-1(violet).

A recent study focused on the establishment of prognostic markers of outcome for acute babesiosis caused by *B*. *canis* [40], though the number of samples in that study was quite low. Since our results point out MCP-1 as the analyte with the most significant prognostic value, we calculated the Receiver Operating Characteristic (ROC) curve (Fig 8) to discriminate uncomplicated from complicated cases on the day 1, with an Area Under the Curve (AUC) of 0.906, indicating an excellent discrimination. ROC curves for individual values of IL-7, GM-CSF and KC-like showed the highest AUC values among the rest of the cytokines in the present study (0.896, 0.867 and 0.729 respectively). Linear combinations of cytokine concentration values yielded higher AUC values that individual cytokines in ROC curves (Fig 8) with highest values obtained corresponding to the combinations including MCP-1, KC-like, IL-7 and GM-CSF (AUC = 0.983for LC3). Cut-off values for every parameter in ROC curves and their corresponding sensitivity and specificities along with AUC values are shown in Table 4.

**Table 4:** Cut-off values (with corresponding True Positive Rate, TPR or sensitivity and False Positive Rate, FPR or 1-specificity) and AUC for cytokines and hematologic parameters. All values correspond to day 1 except HCT. Curve Sensitivity and specificity are indicated for each cut-off value. AUC:Area Under the Curve, SE: Standard Error.

	Cut-off value (pg/ml serum)	Sensitivity (%)	Specificity (%)	AUC	SE
**MCP-1**	360	83.3	84.6	0.906	0.02
**IL-7**	85	83.3	92.3	0.896	0.07
**KC-LIKE**	360	83.3	69.2	0.729	0.11
**GM-CSF**	225	66.7	100	0.867	0.05
**HCT day 7**	42	82.3	69.2	0.757	0.14
**LC1**	1445	83.3	100	0.94	0.07
**LC2**	1520	88.9	100	0.957	0.06
**LC3**	1650	94.4	100	0.983	0.04

Calculation of correlation coefficient was performed for all combinations of cytokines and hematological parameters and the highest values (strongest positive or negative correlations) are summarized in Table 3.

## Discussion

Despite diagnostic and therapeutic advances [41], complications from babesiosis caused by *B*. *canis* continue to be a cause of mortality. Pro-and anti-inflammatory cytokines act in a multifactorial manner after being released following injury, endotoxin release, complement activation and others. In babesiosis, when the inflammatory response becomes uncontrolled, SIRS takes place. SIRS, if not down-regulated, can lead to MODS and, if not treated to terminal condition.

Several efforts have been made in different inlammatory disorders to elucidate the origin of imbalances in cytokine clusters associated to SIRS, MODS and other major complications. TNF- α levels have been correlated with indices of renal damage and blood pressure in dogs infected with *B*. *canis* [42]. Additionally, the injection of TNF-α into experimental animals causes a syndrome resembling septic shock and infusion of recombinant TNF-α into humans results in SIRS [43]. Equilibrium between TNF-α, IL-1β and anti-inflammatory IL-10 has been proven highly dynamic and dependent on genetic heterogenity in human sepsis and SIRS. Our present data registered a global increase of cytokine concentrations in serum of *B*.*canis*-infected dogs though some of them show higher significance, a stronger association with complications and a different longitudinal behaviour. Among the analytes, MCP-1 and KC-like showed significantly different levels in uncomplicated and complicated babesiosis cases on both day 1 and 7. GM-CSF, IL-2 and IL-7 showed significant differences between those same groups but only on day 1. Time-course variation was significant for IL-8 and IL-10 from day 1 to day 7 both in uncomplicated and complicated cases (though with opposite trends as IL-8 increased longitudinally while IL-10 registered a very pronounced decrease). IL-8 showed a negative correlation with erythrocyte counts and hematocrit and a positive correlation with non-segmented neutrophil counts in uncomplicated cases (day 1) whereas KC-like registered as well a negative correlation with erythrocyte counts and hematocrit but in complicated cases (day 7) and a generally strong positive correlation with non-segmented neutrophil counts in all babesiosis groups.

Results observed for MCP-1 were the most significant in this study. MCP-1 together with KC-like are the only cytokines in the present study discriminating complicated from uncomplicated cases both on day 1 and 7. MCP-1 acts in the recruitment of monocytes / macrophages, memory T cells, and dendritic cells to sites where either tissue injury or inflammation occurs [44]. Our results are consistent with previously reported data [45] for human sepsis in which patients show an increased level of MCP-1 with higher concentrations among non-survivors although with no statistically significant differences. Statistically significant differences in MCP-1 levels have been previously found between control dogs (118 pg/mL), survivors (431 pg/mL) and non-survivors (757 pg/mL) in the case of *B*.*canis-* induced babesiosis [18]. Our present data confirm that complicated cases register a higher concentration of MCP-1 than uncomplicated cases and additionally offer a comprehensive insight into the time-dependent variations of this and other cytokines.

MCP-1 has been involved in pathogenesis of several autoimmune diseases characterized by monocytic infiltrates, such as psoriasis, rheumatoid arthritis and atherosclerosis [46]. Some observations have shown that MCP-1 secretion can be induced by erythrocytic debris and coagulopathy [47] the latter being associated to mortality. Paraoxonase 1 (PON-1) has been observed to inhibit this process decreasing endothelial MCP-1 secretion [48] and previous studies have indicated a decrease in PON-1 concentration in acute babesiosis [28]. Moreover, very high plasma levels of MCP-1 and IL-6 have been often observed after incompatible transfusion–induced hemolysis, reinforcing the relationship of immune-mediated hemolysis and MCP-1. In malaria, most of the evidence supports the hypothesis that cells from the monocyte/macrophage lineage are more effective than neutrophils at phagocyting parasitized erythrocytes [31] and this could explain the fact that MCP-1 shows much higher significance as outcome marker than IL-8 in present study.

IL-8 or neutrophil chemotactic factor is secreted as a response of high IL-1 and TNF-α levels, bacterial or viral products and cellular stress [49]. It induces chemotaxis, in neutrophils and other granulocytes. Elevated IL-8 has been documented in many inflammatory conditions for both humans and animals [50, 51] as well as in severe malaria [52] and previously in *B*. *canis*-caused babesiosis [39]. In babesiosis caused by *B*.*rossi*, an exceptional decrease in IL-8 was previously reported [53] probably related to the higher virulence of *B*. *rossi*, pointing out the possibility that a weaker pro-inflammatory response in acute-phase can lead to a worse outcome. Our data show a clear longitudinal increase of IL-8 levels in contrast with data for *B*. *rossi*-caused canine babesiosis [53].

Recent studies found that both IL-8 and MCP-1 levels increase in leptospirosis, another protozoan disease [54]. In turn, IL-10 increased in only 38% of patients at the very early part of diseases, and after 2 weeks it decreased at the level of healthy group. In recent studies about malaria, levels of pro-inflammatory biomarkers, like IL-8, were higher in cerebral malaria (CM) than in non-cerebral malaria patients. In contrast, the concentrations of anti-inflammatory cytokines, like IL-10, were comparable or lower in CM patients [55]. We observed a similar pattern: higher MCP-1 levels in complicated cases and increasing levels of IL-8 in the period studied (day 1 to 7), in remarkable contrast with the decrease observed in *B*. *rossi*-induced babesiosis, and a similar or slightly lower IL-10 concentration associated to complications.

Recent research conducted on cell cultures has proven that the enhanced production of CXC chemokines (IL-8 is an example) can trigger oxidative stress [56] which, besides secondary IMHA, has been considered the main cause of erythrocyte destruction in canine babesiosis [27]. In our data, the negative correlations observed between IL-8 and hematocrit suggests that the sustained increase in IL-8 concentration occurs in parallel to hemolysis. Moreover, in a recent study erythrocyte structure was found to be notably affected by IL-8 with morphological changes resembling those typically observed in eryptosis (programmed red cell death) [57] most probably after interaction with duffy antigen chemokine receptor (DARC) on erythrocyte surface [58]. Additionally, the present data show that IL-8 levels strongly correlate with KC-Like levels among uncomplicated cases in measurements performed on the day 1, as well as in the same group on day 7. Similar results were obtained in a study on canine experimental LPS-induced endotoxemia [59]. Previous studies have shown that the concentration of IL-8 and KC-like are important factors in the pathogenesis of canine babesiosis [39]. A balance between these two cytokines might be important to avoid the occurrence of complications [11]. In relation with other hematologic parameters, we found a strong positive correlation between IL-8 as well as KC-like with non-segmented or immature neutrophils. Shorter life expectancy was recently found in patients with systemic inflammation who showed highest counts of immature neutrophils [60]. The strongest correlation between non-segmented neutrophils and KC-like was registered in complicated cases on day 1, confirming the role of KC-like in neutrophil proliferation. KC-like has been recently reported as a possible biomarker for diagnosing sepsis and uterine bacterial infection in dogs [61]. We found increased levels of KC-like in all diseased groups compared with healthy dogs and most importantly, it discriminates uncomplicated and complicated cases both on day 1 and 7.

IL-10 displays the most significant variation among all the molecules monitored in this analysis when comparing healthy and diseased groups on day 1 of treatment, in agreement with previous data for *B*. *rossi* [53] in which no significant difference in IL-10 was found in non-survivors with respect to survivors. Similarly, we observed no significant differences related to the occurrence of complications but we found a very significant longitudinal drop of IL-10 levels on day 7. IL-10 is an anti-inflammatory cytokine. During infection, IL-10 inhibits Th1 cells, NK cells and macrophages. Given that these cells are required for pathogen clearance, high levels of IL-10 can indirectly contribute to tissue damage. High levels of IL-10 have been detected in mice serum after infection with *B*. *microti* [62]. In that study the frequency of IL-10-producing regulatory B cells (Bregs) and CD4^+^ T cells increased during the course of *B*. *microti* infection. Transfer of IL-10-producing B cells induced by *B*. *microti* infection led to increased susceptibility of recipient mice to infection with *B*. *microti*. In humans with sepsis and early-stage systemic inflammatory response, high levels of IL-10 and IL-10/ TNF-α ratio respectively are linked with poor outcome [63]. IL-10 was observed to be increased in *Plasmodium falciparum*-infected patients in agreement with our study [64] although we observed that IL-10 values reverted to values seen in healthy dogs within 6 days post-diagnosis both in uncomplicated and complicated cases indicating that babesiosis could disrupt the IL-10-producing regulatory B cell mechanisms and the fate of the disease would in part be determined by the initial levels of inflammatory cytokines rather that by the sustained inhibition of the immune reponse.

Our data show a very significant increase in GM-CSF in all diseased groups and higher levels in complicated versus uncomplicated groups on day 1. GM-CSF-deficient mice have been reported to display impaired resistance to blood-stage malaria reflecting the importance of hematopoietic cytokines to fight sepsis-inducing parasites [65]. GM-CSF stimulates stem cells to produce granulocytes (neutrophils, eosinophils, and basophils) and monocytes. Increase in GM-CSF has been reported in *B*.*rossi* infection [53] related to more severe cases.

We observed significant differences between complicated and uncomplicated cases in both IL-2 and IL-7 only on day 1. In the case of *B*. *rossi*- caused babesiosis a similar trend was observed, as well as in human babesiosis caused by *B*. *microti* [66].

Our study shows in summary a general trend in the establisment of a prevalent cytokine cluster led by MCP-1and KC-Like as well as IL-2, IL-7 and GM-CSF and a longitudinal behavior characterised by a steady increase in IL-8 levels together with a decreased concentration of the anti-inflammatory cytokine IL-10. Nevertheless, data must be read cautiously due to the low case numbers and thus the possibility of type 1 error. Another limitation is the heterogenicity in the stage of the disease at the moment of the presentation in clinics as well as the differential treatment in the presence or absence of complications (blood tranfusions for hematocrit values under 20%) and the potential effect on cytokine measurements.

Despite limitations, results obtained in longitudinal study reinforce the trends observed as it confirms the evolution of the inflammatory response in two time points for both uncomplicated and complicated cases. These data suggest the possibility of using cytokine levels as predictors of the occurrence of complications in babesiosis. Individual cytokines and groups of cytokines that might represent outcome-correlated cytokine clusters are receiving much attention in clinical research [64, 67]. The linear combination of 4 cytokines of the present study was found to be the best predictor for the occurrence of complications. Cytokines involved in tissue infiltration might be of major importance when considering the progression from simple hemolysis to SIRS and MODS.

Our data suggest the use of some of the cytokines analysed as prognostic markers. ROC curves for MCP-1 and GM-CSF show a high AUC (area under the curve) for discriminating uncomplicated from complicated cases. Hematocrit values on the day 7 are an interesting parameter to determine outcome and IL-7 shows a similar discriminating power for uncomplicated and complicated cases.

Due to the significance of MCP-1 levels observed in this analysis, a therapeutic approach can be suggested that would inhibit endothelial MCP-1 secretion. Directly affecting MCP-1 expression (using inhitors as thiazolidinedione or anti-inflammatory antibiotics like doxycycline) to inhibit monocyte chemotaxis and migration into organs might result in benefitial effects as observed in studies performed with human lung epithelial cell lines [68] and liver [69]. An easier and more specific possibility is to use antibodies to block MCP-1 activity.

## Conclusion

Complications derived from babesiosis as SIRS and MODS are product of a complex balance of inflammatory and anti-inflammatory signals with an outcome often dependent on genetics, immune status, type of pathogen etc. Thus, clinical practice would greatly benefit from the discovery of tools to predict the onset of complications and therapeutic targets to limit the effects of excessive inflammatory response

Our data show that the hallmark of canine babesiosis caused by *Babesia canis* is a systemic inflammatory response which shares some features with hemolytic disorders and sepsis, thus, both clinical presentation and outcome of disease depend on the balance between pro- and anti-inflammatory response (cytokines). MCP-1 and KC-like discriminate complicated and uncomplicated cases on day 1, representing an important marker for pathology and outcome. IL-8 was found to show a different secretion pattern when compared to published data for babesiosis caused by *B*. *rossi*. This remarkable difference suggests a specific response for less virulent *B*. *canis*, characterised by a clearly increased level of IL-8, this cytokine was found to decrease its levels in *B*.*rossi*-infection, and MCP-1 together with a pronounced longitudinal drop in the level of regulatory IL-10. Consequently, our data suggest a sustained inflammatory response associated to hemolysis that might help in understanding the occurrence of complications and final death of dogs with babesiosis. Present data, as observed in ROC curves, for individual cytokine levels and even clearer for linear combinations of these values, can be useful as a foundation to develop a prognostic tool and explore its performance against a high number of individual samples. Finally, our data suggest as well the possibility of inhibiting the expression and/or secretion of MCP-1 as a way to keep the balance of pro- and anti-iflammatory responses to improve the outcome of canine babesiosis. An easier and more specific possibility is to use antibodies to block MCP-1 activity.

## Supporting information

S1 Table Cytokine levels, hematologic data for all samples and reference ranges used in this study. (XLSX)

## Abbreviations

APTT: Activated partial thromboplastin time
BrEMA1: *Babesia rossi* erythrocyte membrane antigen
CXC chemokines: Cysteine-X-Cysteine motif-containing chemokine
GM-CSF: Granulocyte-macrophage colony stimulating factor
KC-like: Keratinocyte-derived chemokine
IL: Interleukin
IMHA: Immune-mediated hemolytic anemia
MCP-1: Monocyte chemoattractant protein 1
MODS: Multiple organ disfunction syndrome
PCR: Polymerase chain reaction
ROC: Receiver operating characteristic
SIRS: Systemic inflammatory response syndrome
TGF-β: Transforming growth factor beta
PON-1: Paraoxonase-1
